# Some cases of primary hyperparathyroidism may not be truly primary in origin

**DOI:** 10.3389/fendo.2026.1858013

**Published:** 2026-07-07

**Authors:** De-ya Kong, De-ru Kong, Bai-qing Peng, Li-yuan Mu, Xiao-chun Cheng, Xi-rui Li, Xiu-quan Qu, Dong-li Liu, Zhao-hai Li, Ao-ran Li, Ling-quan Kong

**Affiliations:** 1 Xiangya School of Medicine, Central South University, Changsha, China; 2 The First Affiliated Hospital of Chongqing Medical University, Chongqing, China

**Keywords:** calcium and/or vitamin D insufficiency, parathyroid hyperplasia, primary hyperparathyroidism, secondary hyperparathyroidism, tertiary hyperparathyroidism, vitamin D deficiency

## Abstract

Primary hyperparathyroidism (PHPT) is the third most common endocrine disorder and is traditionally considered to result from intrinsic pathology. However, this traditional view is challenged by several persistent clinical and epidemiological observations. Therefore, we hypothesize that some cases of PHPT may not be truly primary in origin. Some of them may be irreversible secondary hyperparathyroidism (tertiary hyperparathyroidism), which may be initiated and perpetuated by widely existed chronic calcium and/or vitamin D insufficiency. The “primary” (in fact, irreversible secondary hyperparathyroidism or “tertiary” hyperparathyroidism) and “secondary” hyperparathyroidism states are not dichotomous but are linked through a process of chronic stimulation, adaptive hyperplasia, and eventual clonal transformation. This model is supported by epidemiological evidence demonstrating an association between low lifelong calcium intake and increased PHPT risk, physiological evidence of persistent PTH suppressibility by calcium intake, and the high rate of clinical misdiagnosis between primary and secondary hyperparathyroidism. If validated, this reframing would mandate a paradigm shift in the understanding and management of PHPT or irreversible secondary hyperparathyroidism. It emphasizes the critical need to rigorously identify and correct underlying nutritional deficiency as a fundamental diagnostic and therapeutic step, transforming the management approach from a predominantly surgical model to one focused on prevention, early medical intervention, and addressing a key modifiable environmental cause

## Introduction

Hyperparathyroidism is a common endocrine disorder worldwide, characterized by excessive secretion of parathyroid hormone (PTH) and disturbances in the body’s calcium and phosphorus metabolism (1). Based on its etiology, hyperparathyroidism is classified into primary hyperparathyroidism (PHPT, due to intrinsic parathyroid pathology), secondary hyperparathyroidism (SHPT, a compensatory response to external stimuli), and tertiary hyperparathyroidism (THPT, following SHPT, the parathyroid glands transform into hyperplastic tissue or adenoma capable of autonomous PTH secretion due to persistent external stimulation) (2). Among these, PHPT is considered the most common type and is the third most common endocrine disorder, with the highest incidence in postmenopausal women (2). The diagnostic and therapeutic paradigm for PHPT has thus been largely surgical, focused on identifying and removing the culprit parathyroid gland(s) (3).

Historically, PHPT has been considered to arise from intrinsic parathyroid pathology in the absence of a known or identifiable external stimulus (2). However, this traditional view is challenged by a series of consistent clinical and epidemiological observations. First, a significant proportion of PHPT patients, including those with normocalcemic variants, were found to have coexisting vitamin D insufficiency or deficiency (4, 5). Second, landmark studies indicated that low dietary calcium intake was a potent, independent risk factor for the development of what is diagnosed as sporadic PHPT (3). Third, evidence showed that parathyroid function in a subset of PHPT patients remains physiologically responsive and can be suppressed by exogenous calcium intake (6, 7). Most strikingly, a notable percentage of patients referred for definitive parathyroid surgery were found to have been misdiagnosed, with the majority actually suffering from SHPT driven by correctable factors like vitamin D deficiency (6). These incongruities suggested a potential continuum between secondary and primary hyperparathyroidism states. Therefore, we hypothesize that some cases of PHPT may not be truly primary in origin. Some of them may be irreversible secondary hyperparathyroidism (tertiary hyperparathyroidism), which may be initiated and perpetuated by widely existed chronic insufficient calcium and/or vitamin D intake.

**Figure 1:**
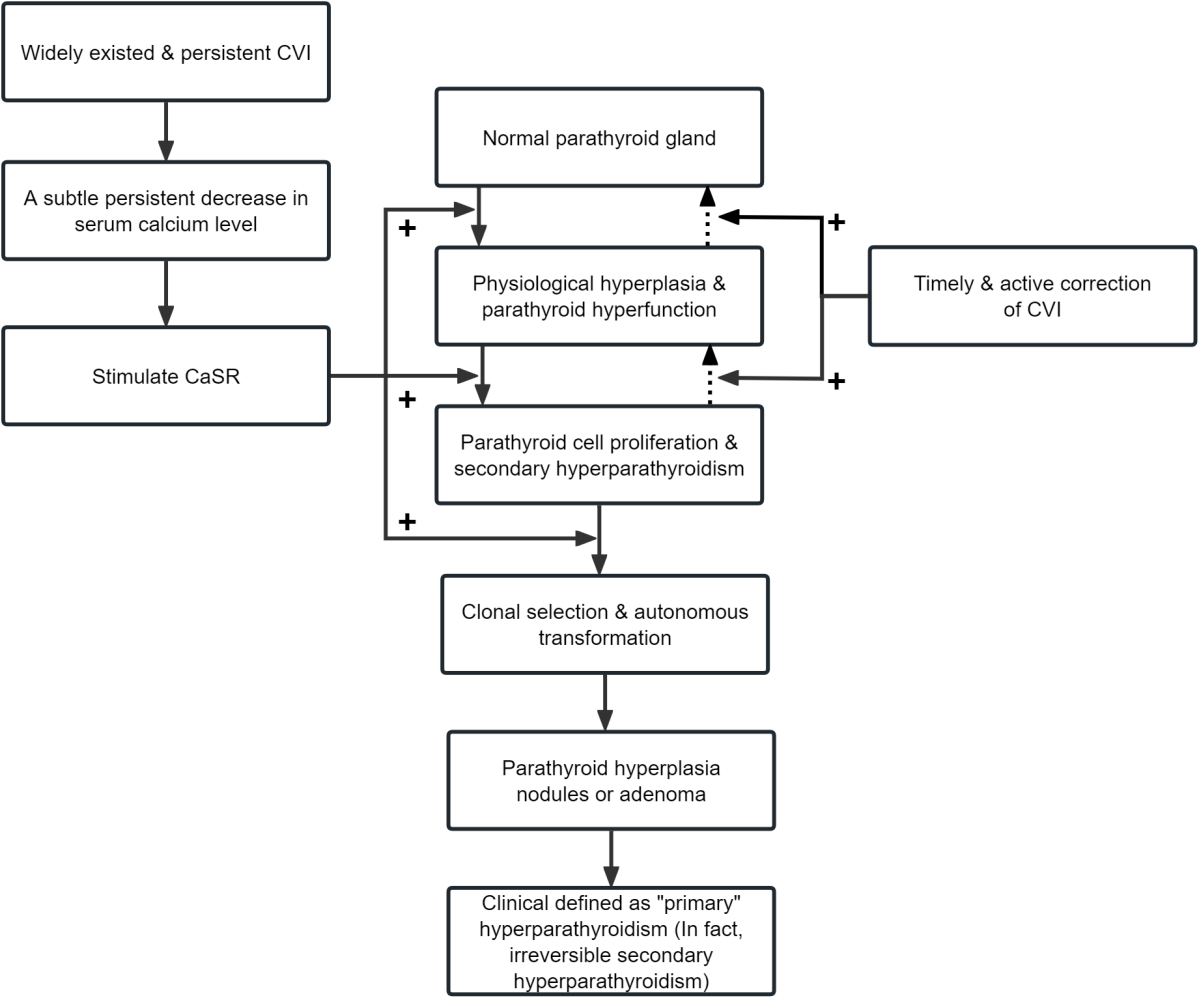
Partial mechanism of some cases of primary hyperparathyroidism may actually not being primary. CVI, calcium and/or vitamin D insufficiency; CaSR, calcium sensing receptor; +: stimulate.

Despite the profound diagnostic and therapeutic implications, this issue has not received adequate attention in clinical practice. Theoretically, certain cases clinically diagnosed as “primary” hyperparathyroidism may represent an advanced and potentially irreversible stage on a pathological continuum, which may be initiated and perpetuated by chronic calcium and/or vitamin D insufficiency. In fact, the “primary” or tertiary (i.e., irreversible secondary hyperparathyroidism, spontaneous, autonomous parathyroid hyperplasia nodules or adenoma) and “secondary” (reactive, physiology-driven hyperplasia) states are not dichotomous but are linked through a process of chronic stimulation, adaptive hyperplasia, and eventual clonal transformation (**Figure 1**). The proposed pathophysiological sequence is as follows.

### Chronic trophic stimulation (phase 1: parathyroid hyperplasia and hyperfunction)

Globally, inadequate calcium intake is highly prevalent (7–9),. And vitamin D deficiency frequently coexists (5, 9). The calcium and/or vitamin D insufficiency may lead to a subtle, persistent decrease in serum calcium, resulting in the hyperplasia, hypertrophy and hyperfunction of parathyroid gland (10). It was proved that changes in the level of PTH can be observed within 20 s as the serum level of ionized calcium decreased. Even a slight decline of 0.025 mmol/L for ionized calcium can lead to significant change in PTH levels (11). As PTH secretion is exquisitely sensitive to ambient calcium levels via the calcium sensing receptor (1), chronic and persistent calcium and/or vitamin D insufficiency provides a constant trophic stimulus. Animal studies confirmed that chronically low calcium intake directly induced parathyroid gland hyperplasia (12, 13). This stage represents physiology-driven parathyroid hyperplasia and hyperfunction, which is potentially reversible upon correction of the initiating calcium and/or vitamin D insufficiency.

### Proliferative drive and genomic instability (phase 2: parathyroid proliferation and secondary hyperparathyroidism)

The prolonged stimulus of calcium and/or vitamin D insufficiency leads to not only increased PTH synthesis and secretion but also enhanced parathyroid cell proliferation (14). Increased cell turnover over years or decades heightens the probability of spontaneous somatic mutations occurring in genes that regulate cell cycle and growth. This prolonged hyperplasia may also lead to an altered calcium set-point and reduced sensitivity of the hyperplastic glands, resulting in non-suppressible PTH secretion even when serum calcium normalizes (e.g., through increased bone resorption). This stage represents pathophysiology-driven parathyroid cell proliferation and secondary hyperparathyroidism. Critically, parathyroid function at this stage may still be partially reversible if the initiating calcium and/or vitamin D insufficiency is aggressively corrected (15, 16). Consistent with this concept, Costa−Guda and colleagues demonstrated in a transgenic mouse model that vitamin D deficiency does not directly initiate parathyroid tumorigenesis but instead promotes the proliferation of parathyroid cells already carrying cyclin D1−driven genetic alterations, supporting the role of nutritional factors as tumor promoters rather than primary initiators (17).

### Clonal selection and autonomous transformation (phase 3: irreversible secondary hyperparathyroidism or tertiary hyperparathyroidism being mistaken as “primary” hyperparathyroidism)

A parathyroid cell that acquires a somatic mutation gains a proliferative advantage. Factors that cause hyperplasia, such as lower calcium intake, increase the probability of such a mutation and subsequent clonal proliferation (3, 18). This mutated clone expands monoclonally, eventually forming a classic parathyroid hyperplasia nodules or adenoma that secrete PTH autonomously (3). This represents the endpoint of the spectrum: histologically and clinically defined irreversible secondary hyperparathyroidism or “**tertiary**” hyperparathyroidism, which may be mistaken as “primary” hyperparathyroidism. The Nurses’ Health Study provided compelling support, showing that higher lifelong calcium intake was associated with a significantly reduced risk (multivariable RR 0.41) of developing “primary” hyperparathyroidism, positioning calcium and/or vitamin D insufficiency as a key modifiable environmental risk factor for what culminates in a neoplastic diagnosis (3).

This continuum hypothesis elegantly explains the frequent finding of vitamin D deficiency in PHPT cohorts (4, 5), the suppressibility of PTH with calcium intake in many patients (15, 16), the high rate of misdiagnosis (6), and the potential for medical management with calcium and vitamin D repletion in selected cases (14, 19).

### Epidemiological and risk factor evidence

The prospective Nurses’ Health Study provides a direct evidence, linking lower dietary and supplemental calcium intake to a higher risk of incident PHPT (3). This is consistent with cross-sectional data from the National Surveys in South Korea, which showed an inverse relationship between calcium intake and PTH levels, particularly in the context of low vitamin D status (20). The global scale of calcium and/or vitamin D insufficiency underscores the vast population at potential risk (7–9).

### Physiological and clinical evidence

Studies demonstrated that parathyroid function in PHPT is not fully autonomous. Calcium supplementation (500–1000 mg/day) can significantly suppress PTH levels and improve bone mineral density in patients with mild PHPT and low baseline intake (15, 16). It was reported that compared with calcium supplementation (1200 mg calcium/day) alone, combined supplementation with vitamin D and calcium (1200 mg calcium plus 800 IU vitamin D3/day) for 8 weeks resulted in a 72% increase in 25-hydroxyvitamin D levels (P< 0.01) and an 17% reduction in PTH levels (P<0.05) (21). A meta-analysis of 26 randomized controlled trials showed that vitamin D_2_ supplementation resulted in a mean reduction in PTH of 12.77 pg/mL and a mean increase in serum calcium of 0.15 mg/dL, suggesting that vitamin D2 supplementation appears to improve serum calcium levels while decreasing PTH levels (22). It was reported that a preoperative calcium challenge was prospectively initiated in normocalcemic patients with parathyroid hormone elevation, and there was high compliance (92%). Short-interval calcium supplementation revealed about 50% have resolved secondary hyperparathyroidism due to insufficient calcium intake, which avoided unnecessary surgery. A short course (e.g., 3–6 months) of calcium and vitamin D supplementation is sometimes used as a “challenge” to see if PTH normalizes, identifying cases caused by insufficient calcium intake rather than a parathyroid tumor. In contrast, classic patients were unveiled in 20%, allowing for prompt and correct surgical intervention (23).

This indicates preserved feedback mechanisms in a substantial patient subset. Furthermore, the high misdiagnosis rate (19% in one surgical series), where most misdiagnosed patients had SHPT from vitamin D deficiency, indicates the clinical blurring between these entities (6).

### Mechanistic and pathophysiological evidence

Animal models have established a causal relationship between low calcium and parathyroid hyperplasia (12, 13). The monoclonal nature of most parathyroid adenomas supports the concept of a clonal outgrowth from a background of hyperplasia (3, 18). The high prevalence of low 25-hydroxyvitamin D in surgical PHPT patients further connects calcium and/or vitamin D insufficiency to the advanced disease state (4).

### Guideline and expert consensus support

It was suggested that all patients with primary hyperparathyroidism, particularly those with low dietary calcium intake, should be advised not to restrict dietary calcium to prevent further increase in PTH levels (24). Current management guidelines for asymptomatic PHPT advise against limiting calcium intake and recommend correcting vitamin D deficiency, acknowledging the non-autonomous component of the disease and the importance of addressing calcium and/or vitamin D insufficiency (14, 19).

## Conclusion

On the basis of clinical and epidemiological observations, we hypothesize that some cases of PHPT may not be truly primary in origin. Some of them may be irreversible secondary hyperparathyroidism, which may be initiated and perpetuated by widely existed chronic calcium and/or vitamin D insufficiency. To validate this hypothesis still requires further scientific research. The following approaches may be helpful: 1) Longitudinal cohort studies: Following large cohorts of individuals with documented biochemical SHPT due to calcium and/or vitamin D insufficiency over decades to quantify the progression rate to hypercalcemic, non-suppressible hyperparathyroidism, and identifying predictive factors. 2) Molecular genetic studies: Comparing the genomic landscape (somatic mutations, clonality) of parathyroid tissue from: a) patients with reversible, calcium and/or vitamin D insufficiency -corrected hyperparathyroidism, b) normocalcemic PHPT, and c) classic hypercalcemic PHPT adenomas. This could identify the “point of no return” in the continuum. 3) Interventional trials: Conducting large-scale, long-term randomized controlled trials to determine if proactive population-level or high-risk individual correction of calcium and/or vitamin D insufficiency can reduce the risk of clinically diagnosed PHPT.

### If validated, this hypothesis would necessitate significant shifts in clinical practice as follows.

Diagnostic re-evaluation: The diagnostic algorithm for PHPT, especially normocalcemic PHPT, should require the rigorous exclusion and active, supervised correction of calcium and/or vitamin D insufficiency before confirming a “primary” diagnosis. A period of optimized calcium and vitamin D repletion should be considered a necessary diagnostic step, not just supportive care.

Therapeutic reorientation: A trial of aggressive, monitored calcium and/or vitamin D insufficiency correction should be the first-line intervention for a significant subset of patients—particularly those with mild/asymptomatic disease, normocalcemia, and evidence of low intake/deficiency—before surgical referral is considered (14, 15).

Preventive focus: Public health strategies should emphasize achieving adequate lifelong calcium and vitamin D intake as a potential primary preventive measure against part of PHPT (8, 25). This is crucial for populations with habitually low dietary calcium.

Acceptance of this continuum hypothesis would fundamentally reframe the understanding of some sporadic PHPT. It moves the paradigm from viewing it predominantly as a spontaneous neoplastic event to recognizing that a significant proportion of cases may have a preventable, metabolic etiology originating in a near-universal environmental exposure (calcium and/or vitamin D insufficiency). It provides a coherent pathophysiological bridge linking secondary, tertiary, and primary hyperparathyroidism.

The most profound consequence is the imperative to transition from a predominantly surgical-reactive model to a medical-preventive and early interventional model. It advocates for the assessment of “calcium and/or vitamin D insufficiency status” to be central, preliminary, and actionable in the evaluation of any elevated PTH. This aligns perfectly with modern paradigms of patient-centered, “all-round and full-cycle” healthcare, aiming to address the root cause.

This model does not negate other etiologies of PHPT, such as those from genetic syndromes or radiation. Furthermore, the threshold at which reversible hyperplasia becomes an irreversible monoclonal adenoma or nodules of parathyroid is likely variable and influenced by individual genetic susceptibility, age, and duration of stimulation. The hypothesis also explains the broader systemic associations of PHPT (e.g., with cardiovascular disease, insulin resistance) (14, 26), as these could be exacerbated by both chronic calcium and/or vitamin D insufficiency and the resulting chronic hyperparathyroidism. In conclusion, this hypothesis, supported by a robust body of evidence, calls for a critical re-appraisal of the “primary” label in irreversible secondary hyperparathyroidism (tertiary hyperparathyroidism). It opens novel avenues for prevention, refined diagnosis, and personalized treatment, potentially reducing unnecessary surgery and improving long-term patient outcomes.

### Limitations of the hypothesis

It should be noted that this theoretical hypothesis currently lacks direct long-term longitudinal verification and targeted molecular mechanistic evidence. While the present framework is grounded in existing published findings, dedicated prospective studies and independent empirical research will be essential to further confirm, refine, or revise the hypothesis in the future.

## Data Availability

The original contributions presented in the study are included in the article/Supplementary Material. Further inquiries can be directed to the corresponding author.
